# Safety and compliance among newly qualified paramedics in a pre-hospital clinical trial of an investigational medicinal product: a post-hoc analysis of the PACKMaN randomised controlled trial

**DOI:** 10.29045/14784726.2025.12.10.3.10

**Published:** 2025-12-01

**Authors:** Andy Rosser, Imogen Gunson, Zoe Green, Ranjit Lall, Felix Michelet, Elisha Miller, Joshua Miller, Hannah Noordali, Gavin Perkins, Owen Stanley, Michael Smyth

**Affiliations:** West Midlands Ambulance Service University NHS Foundation Trust ORCID iD: https://orcid.org/0000-0002-5477-4269; West Midlands Ambulance Service University NHS Foundation Trust ORCID iD: https://orcid.org/0000-0001-8335-3335; West Midlands Ambulance Service University NHS Foundation Trust; University of Warwick; University of Warwick ORCID iD: https://orcid.org/0009-0003-6903-7637; Yorkshire Ambulance Service NHS Trust ORCID iD: https://orcid.org/0000-0003-4729-8572; West Midlands Ambulance Service University NHS Foundation Trust ORCID iD: https://orcid.org/0000-0003-1990-4029; University of Warwick ORCID iD: https://orcid.org/0009-0004-8926-3479; University of Warwick ORCID iD: https://orcid.org/0000-0003-3027-7548; West Midlands Ambulance Service University NHS Foundation Trust ORCID iD: https://orcid.org/0009-0007-9924-4281; University of Warwick ORCID iD: https://orcid.org/0000-0003-0220-2223

**Keywords:** adverse event, clinical trial, compliance, NQP, paramedic, pre-hospital analgesia, research, safety

## Abstract

**Introduction:**

Pre-hospital research has unique challenges. Ambulance clinicians are required to enrol patients in research trials during emergency situations, often remote from the research team at time of recruitment. As newly qualified paramedics (NQPs) represent a significant and growing proportion of ambulance clinicians, it is important to establish whether they can safely and effectively recruit patients to clinical trials. This article reports a post-hoc analysis of the PACKMaN trial, a large, double-blind randomised controlled trial of an investigational medicinal product of ketamine versus morphine in the pre-hospital setting.

**Methods:**

Adverse events (AEs) and serious adverse events (SAEs) experienced by patients recruited to the PACKMaN trial, as well as protocol non-compliances (NCs) experienced by paramedics during the trial, were retrospectively analysed. We compared recruitment, incidence and type of AE, as well as incidence of SAEs and NCs dichotomised by paramedic experience.

**Results:**

Of the 458 patients, 259 (56.6%) and 199 (43.4%) were recruited by experienced paramedics and NQPs, respectively. Incidence of AEs was similar regardless of experience: experienced paramedics reported 128/259 (49.8%) and NQPs reported 91/199 (45.7%) (OR 0.86 95% CI [0.60–1.25]). Incidence of SAEs were slightly increased in the NQP group (8/199 (4.0%)), compared to experienced paramedics (4/259 (1.5%)); however, this was not statistically significant (OR 2.67, 95% CI [0.66–9.00]). NC was similar in both groups: experienced paramedics 3/259 (1.2%) and NQPs 6/199 (3.0%) (OR 2.65 95% CI [0.66–10.74]).

**Conclusion:**

In a double-blind controlled trial of an investigational medicinal product, there was no statistical difference in the incidence of AEs or NCs between NQPs and experienced paramedics. NQPs made an important contribution to patient recruitment in this study, improving the generalisability. SAEs and NCs were rare, and patients received analgesics safely. There was no correlation between experience and AE likelihood, and no safety concerns identified arising from NQP participation. Our findings demonstrate that NQPs can safely recruit patients to clinical trials.

## Introduction

Pre-hospital care has evolved from an emergency transport system to one where appropriately qualified healthcare professionals use their knowledge and skills to deliver high-quality, evidence-based healthcare (Cimino & Braun, 2023; Lowthian et al., 2011; Williams et al., 2009). Pre-hospital clinicians routinely provide the first clinician contact in many healthcare systems, and they play a vital role in helping to reduce morbidity and mortality. In the United Kingdom (UK), pre-hospital care is led by paramedics who are autonomous, professionally registered clinicians, with a title protected in law (Eaton, 2023). The role of the paramedic has evolved significantly since its inception. Today, it is less well-defined and better described as a domain of practice that includes a wide range of skills, knowledge and contribution to research (Williams et al., 2021).

To qualify as a paramedic in the UK, one must first complete a three-year undergraduate degree programme accredited by the Health and Care Professions Council (HCPC). Prior to 2016, the graduate paramedic would have progressed immediately towards an unsupervised lead clinician role (NHS Staff Council, 2017). However, today’s graduate paramedics must undertake a structured newly qualified paramedic (NQP) programme (NHS Employers, 2019). The NQP programme provides a two-year post-qualification foundation period to consolidate skills and knowledge, during which scope of practice may be curtailed, supervised or subject to specific practice restrictions (East of England Ambulance Service, 2024; London Ambulance Service, 2025). It requires that the NQP receives regular supervision and support from senior paramedics and must complete a clinical practice portfolio, including reflective practice and participation in regular scheduled appraisals (College of Paramedics, 2021). This supports the NQP to develop their professional, social and personal identity as a registered paramedic (Phillips & Trenoweth, 2023). Following successful completion of the NQP programme, paramedics are considered sufficiently experienced to no longer require routinely supervised practice.

A large proportion of the NHS ambulance paramedic workforce comprises NQPs, accounting for approximately 20% of the total paramedic workforce at both Yorkshire Ambulance Service NHS Trust (YAS) and West Midlands Ambulance Service University NHS Foundation Trust (WMAS). However, in routine frontline clinical practice, both ambulance services report that a quarter of operational paramedics providing face-to-face contact with patients were NQPs. This may have important implications for the delivery of clinical trials within ambulance services.

It has been reported that there have been fewer high-quality randomised controlled trials (RCTs) completed in ambulance services compared to other healthcare settings and professions (Björklund et al., 2021). This may be attributable to the research culture within ambulance services (Lawrie et al., 2023) and/or the challenging environmental conditions, which reduce the required bandwidth for research activity (Cimino & Braun, 2023). This latter point highlights a potential risk relating to adherence to study protocols and concern for patient safety. Current evidence relating to the participation in research of NQPs and the cognitive burden this might create is sparse. How this may impact research participation by NQPs is unclear. However, there is a plausible risk that inexperienced paramedics may experience bandwidth overload when simultaneously managing clinical and research activity (Copson et al., 2024).

This article aims to explore recruitment, incidence of adverse events (AEs), serious adverse events (SAEs) and protocol non-compliances (NCs) in the Paramedic Analgesia Comparing Ketamine and MorphiNe in trauma (PACKMaN) trial, dichotomised by paramedic experience. The PACKMaN trial was a multi-centre, double-blinded RCT of controlled drugs conducted in two regional NHS ambulance services (Smyth et al., 2025).

## Methods

The PACKMaN trial compared the clinical and cost effectiveness of ketamine and morphine for severe pain following acute traumatic injury and was funded by the National Institute for Health and Care Research Health Technology Assessment (National Institute for Health and Care Research (NIHR), 2024). All study participants were recruited during the period from 10 November 2021 to 16 May 2023.

We conducted a post-hoc analysis of the work experience of paramedics recruiting participants into the PACKMaN trial. This analysis was dichotomised by paramedic experience: experienced paramedics (more than two years post qualification) or NQPs (less than two years qualified). We undertook descriptive analyses of trained paramedics, the patients they enrolled and the AEs, SAEs and NCs for each participating ambulance service relating to the PACKMaN trial recruitment. A chi-square test of independence was used to determine the association between variables.

This post-hoc analysis was conducted using the PACKMaN trial data, without *a priori* intent within the study protocol, and expands upon a mid-point analysis requested by the trial steering committee. The analysis was requested to provide assurance of safety with NQP participation, during the participant recruitment phase of the trial. This post-hoc analysis includes all 458 patient participants and all 489 paramedic participants.

### Setting

PACKMaN was undertaken in WMAS and YAS. WMAS serves a population of 5.6 million over a geographical area of 5500 square miles (West Midlands Ambulance Service, 2024), while YAS serves five million people over 6000 square miles (Yorkshire Ambulance Service, 2024). Collectively, these services receive more than two million 999 emergency calls annually and employ more than 14,000 staff. WMAS employs more than 2700 paramedics, with 88% of these routinely providing clinical response to 999 calls. Of these paramedics, 25.2% are NQPs. YAS employs almost 2200 paramedics, of whom 22.8% are NQPs routinely providing clinical response to 999 calls.

### Participants

All registered paramedics at participating ambulance stations, regardless of prior experience, were eligible to participate in the PACKMaN trial. We dichotomised participating paramedics into NQP and experienced groups. At the time of undertaking training for PACKMaN, paramedics who were undertaking their NQP phase were categorised as NQP, while paramedics who had successfully completed their NQP phase were categorised as experienced.

All participating paramedics, irrespective of experience, received a bespoke training package to ensure understanding of the trial procedures, pharmacology of trial medicines and outcome reporting. Training was delivered either through online or face-to-face delivery. Naloxone hydrochloride and midazolam hydrochloride were available to participating paramedics as open-label medicines in the event of opioid-induced respiratory depression or ketamine-induced hallucinations. All participating paramedics were required to complete a patient group directive to supply or administer the investigational medicinal product and midazolam, in accordance with the study protocol.

### Patient and public involvement

Patient and public involvement (PPI) was embedded into the PACKMaN trial throughout. PPI featured at all stages, through design, study materials, conduct and dissemination. We obtained broad representation through those with personal experience of severe trauma and specialist PPI groups.

### Outcomes

During the PACKMaN trial all AEs, SAEs and NCs were recorded and analysed; findings were reported to the independent data monitoring committee. We linked each of these events to the participating paramedic to assess if their prior level of experience might have been a contributing factor.

AEs and SAEs were pre-specified and well defined, and were reported by recruiting clinicians, hospital clinicians or study research teams as soon as they were identified. Safety reporters were blinded to the study arm and to the paramedic group (NQP or experienced).

An AE was defined as any untoward medical occurrence in a patient administered a medicinal product. Predictable drug side effects were recorded as AEs. An SAE is an AE that fulfils one or more of the following criteria:

- Results in death
- Is immediately life-threatening
- Requires hospitalisation or prolongation of existing hospitalisation
- Results in persistent or significant disability or incapacity
- Causes a congenital abnormality or birth defect
- Requires immediate intervention to prevent one of the above or is otherwise an important medical condition.

Non-compliance was defined as deviation from the trial protocol or trial-related procedures. Non-compliance was not limited to clinician‒patient interaction. For example, failure to complete case report forms could be a non-compliance event. In this analysis we have reported NCs attributable to participating paramedics only; we have not reported NCs attributable to other causes (e.g. trial team staff).

We report descriptive statistics, Fisher’s exact test, chi square and logistic regression to explore the incidence and significance (p = <0.05) of recruitment, AEs, SAEs and NCs dichotomised by paramedic experience (NQP versus experienced paramedic).

## Results

In total, 489 paramedics completed the training necessary to recruit patients to PACKMaN. Participating paramedics were more likely to be experienced paramedics (66.9%) than NQPs (33.1%). There was no significant difference in participation between the two ambulance services (Table 1). During the study, experienced paramedics recruited 56.6% of patients while NQPs recruited 43.4%. NQPs in YAS recruited a greater proportion of patients than NQPs in WMAS did (Table 1).

**Table 1.** Experience and recruitment by participating paramedics.

Number of paramedics^a^	WMAS n (%)	YAS n (%)	Total n (%)
Experienced paramedic	171 (69.5)	156 (64.2)	327 (66.9)
NQP	75 (30.5)	87 (35.8)	162 (33.1)
**Recruitment by paramedic** ^b^			
Experienced paramedic	149 (64.0)	110 (48.9)	259 (56.6)
NQP	84 (36.0)	115 (51.1)	199 (43.4)

^a^ Chi square (1, *N* = 489) = 1.5586, *p* = 0.212

^b^ Chi square (1, *N* = 458) = 10.5652, *p* = 0.001

PACKMaN employed a double-blind randomisation strategy to conceal the medication administered from both paramedics and patients. Our analysis by treatment arms indicates that there was no significant difference in the number of patients recruited to each arm of the study across paramedic experience levels at either site, nor was there any difference in the study arm randomisation according to experience level (Table 2).

**Table 2.** Recruitment by trial arm.

Recruitment by service^a^	Morphine n (%)	Ketamine n (%)	Total n (%)
YAS	113 (48%)	112 (50%)	225
WMAS	121 (52%)	112 (50%)	233
**Recruitment by experience** ^b^			
NQP	106 (45.3%)	93 (41.5%)	199 (43.4%)
Experienced paramedic	128 (54.7%)	131 (58.5%)	259 (56.6%)

^a^ Chi square (1, *N* = 458) = 0.1338, *p* = 0.715

^b^ Chi square (1, *N* = 458) = 0.666, *p* = 0.414

Throughout the study period, 219 patients (47.8%) experienced at least one AE, 12 patients (2.6%) suffered SAEs and there were 10 NC events involving nine patients (2.0%) (Table 3). Some patients experienced more than one adverse event simultaneously, for example experiencing both respiratory depression and hypotension at the same time. Cumulative reported adverse events by clinical category are reported in Table 4. The vast majority of AEs were clinically predictable side effects of morphine or ketamine*.* There was no difference in occurrence of AEs among paramedics attributable to level of experience [OR 0.86 (0.60 to 1.25); p = 0.43].

**Table 3.** Safety-related events by paramedic experience level.

Safety events by experience	Experienced paramedic (n = 259)	NQP (n = 199)	Unadjusted odds ratio (OR) (95% CI); p-value
Adverse events, n (%)	128 (49.4%)	91 (45.7%)	OR 0.86 (0.60 to 1.25); 0.43
Serious adverse events, n (%)	4 (1.5%)	8 (4.0%)	OR 2.67 (0.79 to 9.00); 0.10
Non-compliances, n (%)	3 (1.2%)	6 (3.0%)	OR 2.65 (0.66 to 10.74); 0.16

**Table 4.** Number of adverse events by category, split by trial arm and paramedic experience level*.*

	Trial arm	Airway (n)	Respiratory (n)	Cardiovascular (n)	Neurological (n)	Other (n)
NQP	Morphine	13	18	22	10	24
Experienced paramedic	Morphine	14	20	12	8	33
NQP	Ketamine	9	7	10	18	14
Experienced paramedic	Ketamine	7	8	16	24	20

Of the 12 SAEs, four occurred among experienced paramedics and eight among NQPs (Table 5). Despite the apparent increased frequency of SAE among NQPs, the difference was not statistically significant [OR 2.67 (0.79 to 9.00); p = 0.10]. Of the 10 NC events, three were related to patient recruitment by experienced paramedics and seven were related to patient recruitment by NQPs (see Table 3). Again, this difference was not statistically significant [OR 2.65 (0.66 to 10.74); p = 0.16].

**Table 5.** Serious adverse events and non-compliance.

Serious adverse events by paramedic experience^a^	Morphine (n)	Ketamine (n)	Total (n)
NQP	6	2	8
Experienced paramedic	2	2	4
**Non-compliance by paramedic experience** ^b^			
NQP	5	2	7
Experienced paramedic	2	1	3

^a^ Chi square (1, *N* = 12) = 0.7500, *p* = 0.386

^b^ Chi square (1, *N* = 10) = 0.0227, *p* = 0.880

Similarly, when we examined SAEs and NCs by trial arm, we failed to identify any statistically significant difference attributable to level of experience (Table 5).

## Discussion

In this analysis of data from the PACKMaN trial, we found that NQPs did not experience a higher incidence of AEs, SAEs or NCs compared to their more experienced paramedic colleagues. This suggests that participation of NQPs in clinical research does not increase the risk of safety-related events for patients enrolled in clinical trials. We found no evidence of safety implications related to the inclusion of NQPs in this trial. Consequently, NQPs should not be discouraged from participating in research on the grounds of patient safety.

Our analysis is supported by findings from other clinical trials conducted in UK ambulance services. Clinical trials of devices, including PARAMEDIC (Perkins et al., 2015), AIRWAYS-2 (Benger et al., 2018) and ACUTE (Fuller et al., 2020), and clinical trials of investigational medicinal products (CTIMP), such as PARAMEDIC2 (Perkins et al., 2018) and RIGHT-2 (RIGHT-2 Investigators, 2019), have permitted participation by NQPs and have also reported low incidence of AEs and SAEs.

In the PACKMaN trial, approximately one-third of participating paramedics were NQPs. This proportion is broadly reflective of the experience mix seen in clinical practice across ambulance services more generally. The inclusion of NQPs in the PACKMaN trial also reflects the pragmatic nature of ambulance services, with respect to both participation in research and the introduction of new medicines. NQPs comprise a significant proportion of the operational workforce. Inclusion of NQPs in clinical research is therefore more representative of workforce capability; findings will consequently be more generalisable. Furthermore, if ketamine is added to the paramedic formulary, then NQPs may be expected to use it safely. Inclusion of NQPs in PACKMaN has helped to establish that ketamine can be used safely by NQPs.

We recognise that the paramedics who volunteered to participate in the PACKMaN trial may not truly be representative of the wider paramedic workforce, and we did not record the motivating reason why some paramedics choose to participate. Nonetheless, we are hopeful that these research findings would be generalisable to the wider paramedic workforce, including the NQP workforce. Furthermore, we would caution that findings from clinical research studies that exclude NQPs may not be generalisable to the UK ambulance service clinical workforce.

It is also important to acknowledge that NQPs may play a vital role in recruitment to clinical trials. In PACKMaN, NQPs were responsible for recruiting 43.4% of participants. Without NQP participation, recruitment would have taken considerably longer, incurring substantial and avoidable costs. Excluding NQPs from participation in clinical trials without robust justification may render clinical trials in ambulance services financially unviable. Failure to undertake research may unintentionally extend the provision of sub-optimal treatments, leading to increased harm or suffering.

It has been suggested that when managing complex or severely injured patients, NQPs may experience high levels of stress and be close to being overwhelmed. In such cases, it may be inappropriate to expect NQPs to undertake research activities, when they might be struggling to manage a complex clinical case. This argument asserts that NQPs have limited bandwidth and that research activities consume scarce bandwidth, potentially to the detriment of both the NQP and patient care (Lawrie et al., 2023). We believe our findings refute this argument; we found no evidence of safety implications regarding the inclusion of NQPs in this trial. However, we acknowledge that these claims must not be overstated, and in the presence of sparse and potentially conflicting evidence, it is clear that further research is required, exploring the impacts of cognitive load in NQPs.

Although ketamine is used routinely by non-specialist paramedics in Australia, New Zealand, South Africa, the USA, Canada and several European countries, in the UK its use has been restricted to critical care and specialist paramedics, where its primary indications include analgesia for severe pain that has proved unresponsive to morphine and procedural sedation. The inclusion of ketamine for non-specialist paramedics was always likely to provoke emotive views in some communities, further heightened by the involvement of NQPs. Some services mandate that ketamine administration requires anaesthetic-level monitoring, and clinicians must have advanced airway management capability (Aldridge et al., 2023; Morgan et al., 2021).

In the PACKMaN trial, patients received routine non-invasive physiological monitoring, and paramedics did not use end-tidal carbon dioxide (EtCO_2_) monitoring or require the presence of clinicians competent to perform endotracheal intubation (ETI). Despite a lower level of physiological monitoring and a lack of ETI capability, patients did not experience an increased incidence of AEs (Smyth et al., 2025). Furthermore, as we have already reported, there was no significant difference in AEs attributable to experience. Ketamine therefore appears to be safe in the hands of NQPs providing sub-dissociative pain relief. Excluding NQPs from research participation would have limited the generalisability of the study to UK practice, where NQPs are an important group of the ambulance workforce.

Finally, even though NQPs are undertaking a development pathway, as HCPC registrants they are still accountable to the professional body and held to the same standard as their fellow registrants. Consequently, there is a professional expectation that NQPs are able to provide the same standard of care expected of a more experienced paramedic. We are grateful to our NQPs who took part in this study. They made an important contribution, not only in terms of patient enrolment but also in improving the generalisability of study findings across the ambulance setting. We recommend that the default approach in pre-hospital research should be to include NQPs.

### Limitations

Our study has four notable limitations. First, the study required NQPs and paramedics to volunteer to participate in the PACKMaN trial. The reason why individual NQPs or experienced paramedics opted to participate ‒ or, more importantly, not participate ‒ in the study was not captured. We did not record their confidence in managing patients with traumatic injury, confidence in delivering potent analgesics or confidence to participate in a CTIMP. We did not measure the cognitive impact of NQPs and paramedics recruiting patients to a controlled drug CTIMP. It is therefore unclear how these study findings relate to routine NQP and paramedic practice within a wider workforce, and there is opportunity for further research to understand those cognitive loads.

Second, the data presented formed a post-hoc analysis of the PACKMaN study data; we did not precisely capture the experience of participating paramedics. For example, an NQP who had attended university straight from school was considered the same as an NQP who had 10 years of experience as an ambulance technician prior to attending university. Similarly, we dichotomised paramedics at the time they completed trial-related training. Paramedics who transitioned from NQP to experienced status after they had completed trial training were still classified as NQP. We did not collect data relating to progress in the NQP programme, and we are therefore unable to describe how NQPs were distributed across the two-year period.

Third, PACKMaN was conducted in WMAS and YAS only; therefore, the generalisability to NQP participation in research within other UK ambulance services is not certain. We are buoyed by infrequent rates of adverse event and protocol non-compliance within two large regional ambulance services and see no reason why a larger cohort of participating paramedics might adversely impact that occurrence.

Fourth, the findings of this study may not be transferable to international settings due to training, education and professional differences from UK paramedics.

## Conclusion

The incidence of AEs, SAEs and NCs in PACKMaN, a CTIMP of controlled drugs, was low. We found no evidence to suggest that experience of the participating paramedic was a potential contributing factor to AEs, SAEs or NCs. Our findings further demonstrate that NQPs recruited almost half of all patients participating in the PACKMaN trial, suggesting that NQPs may have a vital role to play in ensuring clinical trials recruit to target in a timely manner. Finally, because NQPs comprise around one-third of the operational workforce in UK ambulance services, inclusion of NQPs in research will significantly improve generalisability of research findings to real-world clinical practice. We recommend that NQPs should be encouraged to participate in research.

## Acknowledgements

We are indebted to the patients that agreed to participate in the PACKMaN trial. We reserve special thanks for each of the paramedics that participated in the trial at both WMAS and YAS.

## Author contributions

All authors made substantial contribution to the data processing, interpretation and production of the article. Their contributions extended to reviewing and revising the article to ensure accuracy. MAS conceived and was the co-chief investigator, with GDP, of the PACKMaN trial. MAS and HN managed the PACKMaN trial centrally. FM provided data analysis and statistical support, with oversight from RL. AR, IG and JM managed the study at WMAS. OS, ZG, EM and JM contributed to data collection. EM managed the study at YAS. MS acts as the guarantor for this article.

## Conflict of interest

None declared.

## Ethics

West of Scotland REC 1, reference 20/WS/0126, approval granted 1 September 2020. Written informed consent was obtained from all participants, according to the study protocol (Michelet et al., 2023).

## Funding

The study was independently peer reviewed as part of the funding application to the National Institute for Health and Care Research, Health Technology Assessment (NIHR128086).

## References

[bibr_1] Aldridge J. Quinn S. A. Oh S. et al (2023). Ketamine for pre-hospital analgesia and sedation in the trauma population: A narrative review. *SN Comprehensive Clinical Medicine*, 5(1), 63. https://doi.org/10.1007/s42399-023-01409-z.

[bibr_2] Benger J. R. Kirby K. Black S. et al (2018). Effect of a strategy of a supraglottic airway device vs tracheal intubation during out-of-hospital cardiac arrest on functional outcome: The AIRWAYS-2 randomized clinical trial. *Journal of the American Medical Association*, 320(8), 779–791. https://doi.org/10.1001/jama.2018.11597. 30167701 10.1001/jama.2018.11597 PMC6142999

[bibr_3] Björklund M. K. Cruickshank M. Lendrum R. A. et al (2021). Randomised controlled trials in pre-hospital trauma: A systematic mapping review. *Scandinavian Journal of Trauma, Resuscitation and Emergency Medicine*, 29(1), 65. https://doi.org/10.1186/s13049-021-00880-8. 34001219 10.1186/s13049-021-00880-8 PMC8127177

[bibr_4] Cimino J., & Braun C. (2023). Clinical research in prehospital care: Current and future challenges. *Clinical Practice*, 13(5), 1266–1285. https://doi.org/10.3390/clinpract13050114. 10.3390/clinpract13050114 PMC10605888 37887090

[bibr_5] College of Paramedics. (2021). *Scope of practice*. College of Paramedics. Retrieved December 10, 2024, from https://collegeofparamedics.co.uk/COP/ProfessionalDevelopment/Scope_of_Practice.aspx.

[bibr_6] Copson J. Eaton G., & Mahtani K. R. (2024). Transition processes for newly qualified paramedics entering primary care: A critical discussion and theoretical perspective. *British Journal of General Practice*, 74(742), 228–231. https://doi.org/10.3399/bjgp24X737337. 10.3399/bjgp24X737337 PMC11060825 38664045

[bibr_7] East of England Ambulance Service. (2024). *Scope of practice policy – POL092*. East of England Ambulance Service NHS Trust. Retrieved September 20, 2025, from https://content.eastamb.nhs.uk/assets/Scope_of_Practice_Policy_84cfabb7f9.pdf.

[bibr_8] Eaton G. (2023). Addressing the challenges facing the paramedic profession in the United Kingdom. *British Medical Bulletin*, 148(1), 70–78. https://doi.org/10.1093/bmb/ldad024. 37681285 10.1093/bmb/ldad024 PMC10724452

[bibr_9] Fuller G. Keating S. Goodacre S. et al (2020). Is a definitive trial of prehospital continuous positive airway pressure versus standard oxygen therapy for acute respiratory failure indicated? The ACUTE pilot randomised controlled trial. *BMJ Open*, 10(7), e035915. https://doi.org/10.1136/bmjopen-2019-035915. 10.1136/bmjopen-2019-035915 PMC7380855 32709643

[bibr_10] Lawrie L. Duncan E. M. Lendrum R. et al (2023). Challenges and opportunities for conducting pre-hospital trauma trials: A behavioural investigation. *Trials*, 24(1), 157. https://doi.org/10.1186/s13063-023-07184-5. 36864520 10.1186/s13063-023-07184-5 PMC9983243

[bibr_11] London Ambulance Service. (2025). *Newly qualified paramedic roles – FAQs*. Retrieved July 30, 2025, from https://www.londonambulance.nhs.uk/working-for-us/career-opportunities/newly-qualified-paramedic-roles-faqs/.

[bibr_12] Lowthian J. A. Cameron P. A. Stoelwinder J. U. et al (2011). Increasing utilisation of emergency ambulances. *Australian Health Review*, 35(1), 63–69. https://doi.org/10.1071/ah09866. 21367333 10.1071/AH09866

[bibr_13] Michelet F. Smyth M. Lall R. et al (2023). Randomised controlled trial of analgesia for the management of acute severe pain from traumatic injury: Study protocol for the paramedic analgesia comparing ketamine and morphine in trauma (PACKMaN). *Scandinavian Journal of Trauma, Resuscitation and Emergency Medicine*, 31(1), 84. https://doi.org/10.1186/s13049-023-01146-1. 38001541 10.1186/s13049-023-01146-1 PMC10668487

[bibr_14] Morgan M. M. Perina D. G. Acquisto N. M. et al (2021). Ketamine use in prehospital and hospital treatment of the acute trauma patient: A joint position statement. *Prehospital Emergency Care*, 25(4), 588–592. https://doi.org/10.1080/10903127.2020.1801920. 32776812 10.1080/10903127.2020.1801920

[bibr_15] National Institute for Health and Care Research (NIHR). (2024). *A randomised controlled trial of Paramedic Analgesia Comparing Ketamine and MorphiNe in trauma (PACKMaN)*. National Institute for Health and Care Research. Retrieved December 13, 2024, from https://fundingawards.nihr.ac.uk/award/NIHR128086.

[bibr_16] NHS Employers. (2019). *Paramedic consolidation of learning*. NHS Employers. https://www.nhsemployers.org/articles/paramedic-consolidation-learning.

[bibr_17] NHS Staff Council. (2017). *National profiles for ambulance service*. NHS Employers. https://www.nhsemployers.org/sites/default/files/2021-06/Ambulance-services-profile.pdf.

[bibr_18] Perkins G. D. Ji C. Deakin C. D. et al (2018). A randomized trial of epinephrine in out-of-hospital cardiac arrest. *New England Journal of Medicine*, 379(8), 711–721. https://doi.org/10.1056/NEJMoa1806842. 30021076 10.1056/NEJMoa1806842

[bibr_19] Perkins G. D. Lall R. Quinn T. et al (2015). Mechanical versus manual chest compression for out-of-hospital cardiac arrest (PARAMEDIC): A pragmatic, cluster randomised controlled trial. *Lancet*, 385(9972), 947–955. https://doi.org/10.1016/s0140-6736(14)61886-9. 25467566 10.1016/S0140-6736(14)61886-9

[bibr_20] Phillips P., & Trenoweth S. (2023). Crossing the ‘flaky bridge’ – the initial transitory experiences of qualifying as a paramedic: A mixed-methods study. *British Paramedic Journal*, 8(1), 18–27. https://doi.org/10.29045/14784726.2023.6.8.1.18. 10.29045/14784726.2023.6.8.1.18 PMC10240861 37284606

[bibr_21] RIGHT-2 Investigators. (2019). Prehospital transdermal glyceryl trinitrate in patients with ultra-acute presumed stroke (RIGHT-2): An ambulance-based, randomised, sham-controlled, blinded, phase 3 trial. *Lancet*, 393(10175), 1009–1020. https://doi.org/10.1016/s0140-6736(19)30194-1. 30738649 10.1016/S0140-6736(19)30194-1 PMC6497986

[bibr_22] Smyth M. A. Noordali H. Starr K. et al (2025). Paramedic analgesia comparing ketamine and morphine in trauma (PACKMaN): A randomised, double-blind, phase 3 trial. *The Lancet Regional Health – Europe*, 53, 101265. https://doi.org/10.1016/j.lanepe.2025.101265. 40247853 10.1016/j.lanepe.2025.101265 PMC12002782

[bibr_23] West Midlands Ambulance Service. (2024). *About us*. West Midlands Ambulance Service University NHS Foundation Trust. Retrieved December 10, 2024, from https://www.wmas.nhs.uk/about/.

[bibr_24] Williams B. Beovich B., & Olaussen A. (2021). The definition of paramedicine: An international Delphi study. *Journal of Multidisciplinary Healthcare*, 14, 3561–3570. https://doi.org/10.2147/jmdh.S347811. 35002246 10.2147/JMDH.S347811 PMC8722816

[bibr_25] Williams B. Onsman A., & Brown T. (2009). From stretcher-bearer to paramedic: The Australian paramedics’ move towards professionalisation. *Australasian Journal of Paramedicine*, 7, 1–12.

[bibr_26] Yorkshire Ambulance Service. (2024). Yorkshire Ambulance Service NHS Trust. Retrieved December 10, 2024, from https://www.yas.nhs.uk/about-us/about-us/.

